# Urban Heat Island Monitoring and Impacts on Citizen’s General Health Status in Isfahan Metropolis: A Remote Sensing and Field Survey Approach

**DOI:** 10.3390/rs12081350

**Published:** 2020-04-24

**Authors:** Mohsen Mirzaei, Jochem Verrelst, Mohsen Arbabi, Zohreh Shaklabadi, Masoud Lotfizadeh

**Affiliations:** 1 Environmental Pollutions, Grape Environmental Science Department, Research Institute for Grapes and Raisin (RIGR), Malayer University, Malayer 65719-95863, Iran; 2 Image Processing Laboratory (IPL), Parc Científic, Universitat de València, Paterna, 46980 València, Spain; 3 Department of Environmental Health Engineering, School of Health, Shahrekord University of Medical Sciences, Shahrekord 88157-13471, Iran; 4 Social Determinants of Health Research Center, Shahrekord University of Medical Sciences, Shahrekord 88157-13471, Iran

**Keywords:** urban heat island, land surface temperature, split window algorithm, general health questionnaire-28, Isfahan metropolis

## Abstract

Urban heat islands (UHIs) are one of the urban management challenges, especially in metropolises, which can affect citizens’ health and well-being. This study used a combination of remote sensing techniques with field survey to investigate systematically the effects of UHI on citizens’ health in Isfahan metropolis, Iran. For this purpose, the land surface temperature (LST) over a three-year period was monitored by Landsat-8 satellite imagery based on the split window algorithm. Then, the areas where UHI and urban cold island (UCI) phenomena occurred were identified and a general health questionnaire-28 (GHQ-28) was applied to evaluate the health status of 800 citizens in terms of physical health, anxiety and sleep, social function, and depression in UHI and UCI treatments. The average LST during the study period was 45.5 ± 2.3 °C and results showed that the Zayandeh-Rood river and the surrounding greenery had an important role in regulating the ambient temperature and promoting the citizens’ health. Citizens living in the suburban areas were more exposed to the UHIs phenomena, and statistical analysis of the GHQ-28 results indicated that they showed severe significant (*P* < 0.05) responses in terms of non-physical health sub-scales (i.e., anxiety and sleep, social functioning, and depression). Therefore, it can be concluded that not all citizens in the Isfahan metropolis are in the same environmental conditions and city managers and planners should pay more attention to the citizens living in the UHIs. The most important proceedings in this area would be the creation and development of parks and green belts, as well as the allocation of health-medical facilities and citizen education.

## 1 Introduction

Over the last decades, global warming has threated human and environment health [1–4]. At the same time, urbanization and urban activities can exacerbate the effects of global warming, as more populations are exposed to heating effects [5,6]. Human activities in changing natural land covers can lead to changes in thermal capacities, albedo coefficient, heat conductivity, and moisture [7–9]. Urban land use can cause the local air and surface temperatures to increase several degrees higher than the temperatures of the surrounding environment [10–12]. This phenomenon is often referred to as an urban heat island (UHI), which has been documented since Howard [13].

With the rise of urbanization and the formation of metropolises, the UHI shows more impact on humans and living environments and supports a wide range of environmental changes in cities, i.e., ecosystem services and functions and local weather and micro-climates [14–16]. In fact, in many previous studies the occurrence of the UHI phenomenon was considered as one of the most important problems of overheating in urban areas (e. g., Li et al. [17] in Shanghai, Enete et al. [18] in Douala, Umar et al. [19] in Kano, and de Faria Peres et al. [20] in Rio de Janeiro).

The UHI can be identified by earth surface temperatures [14]. Nowadays, many satellite sensors can be used to monitor and evaluate earth characteristics. Among these, some satellite data are more commonly used in UHI monitoring, such as MODIS [21,22], ASTER [15], and Landsat TM, ETM^+^ and OLI/TIRS [15,23,24]. Landsat-8 with OLI and TIRS sensors at sufficiently high and medium spatial resolutions (equal to 30 and 100 m, respectively) proved to be particularly suitable for observing land surface temperature (LST) at city scale [15,25]. According to Mohamed et al. [25], the usage of two separate, relatively narrow thermal bands has been shown to minimize error in the retrieval of LST. Therefore, Landsat-8 that is equipped with two thermal infrared channels in the atmospheric window provides a new LST retrieval opportunity based on the widely used split-window algorithm (SWA) rather than the single-channel method [25–27].

Increased temperature adds extra pressure to human physiology and makes the body more vulnerable to stresses. Residents living in a UHI region, or in its vicinity, are at increased health risk [28]. Therefore, the UHI is one of the major urban planning problems, which requires monitoring as well dedicated strategies to reduce its impact [29]. As an example, Gosling et al. [30] reported that an increasing urban temperature has a direct impact on the increase in mortality rate. Other studies have shown that citizens in UHI areas suffer with heat-related illnesses, e.g., digestive diseases, nervous system issues, insomnia, depression, and mental illnesses [31,32]. UHIs have also directly led to a rise in infectious diseases [33]. Along with this, in a systematic review by Thompson et al. [34] increased risks of mental health-related admissions and emergency department visits in higher temperatures areas were found. The findings of Jenerette et al. [35] also demonstrated that the symptoms of heat-related illness were correlated with parcel-scale surface temperature patterns during the daytime in an urban ecosystem. More psychological and social health issues associated to UHI, such as depression and the restriction of social activities, were further reported by Wong et al. [36].

Therefore, when aiming to evaluate the effect of UHIs on the health of citizens a simple tool is required that can easily assess the health status of citizens. Using a questionnaire to assess the health state is a rapid, low-cost, common, and practical approach which can be applied on a large scale (such as in a metropolis) [37–39]. In this regard, the general health questionnaire-28 (GHQ-28) is one of the most commonly used tools for assessing physical and mental health worldwide and has multiple, self-administered constructs. The method was developed by Goldberg [40] and has a 28-item measure of emotional distress in medical settings [41]. The GHQ-28 has been tested in numerous populations including people with stroke [42], spinal cord injury [43], heart disease [44], various musculoskeletal conditions including whiplash associated disorders [45], and occupational lower back pain [46], amongst others. Thus, for clinicians, there is a wealth of data with which to relate patient outcomes [41].

Isfahan is the third largest metropolis in Iran. The city has grown substantially in population and industrialization over the recent decades. The occurrence and spatial pattern of the UHI in Isfahan in 2013–2015 was earlier investigated by Ahmadi and Roudbari [47]. Their results showed that there is a sharp temperature difference among the various regions of the city. This extreme heat variance in different regions of the city can lead to disturbances in the citizen’s health parameters. In other words, our main question was whether citizens of the heat island show an increased health risk. Assessing the relation between the UHI and human general health status is an important step in order to provide information for future planning projects, plans, programs, and policies contributing to public health protection in Isfahan metropolis.

To this end, the first step to take in examining its effects on citizen’s health is determining regions where heat and cold islands occur. Secondly, based on the identified heat map, it should lead to a benchmark that can quantify the different general health sub-scales (physical health, anxiety and sleep, social function, and depression) of the citizens in the heat and cold island regions. Therefore, in order to identify the location of the UHI and urban cold island (UCI) in Isfahan the Landsat-8 satellite data with a SWA were used because of their good adaptation and high performance in LST estimation and UHI monitoring [25,26]. After estimating the LST in Isfahan and the spatial-temporal analysis of cold and hot islands in the city, the health status of the citizens related to life in UHIs and UCIs was assessed using a GHQ-28.

## 2 Materials and Methods

### 2.1 Study area

**Figure 1:**
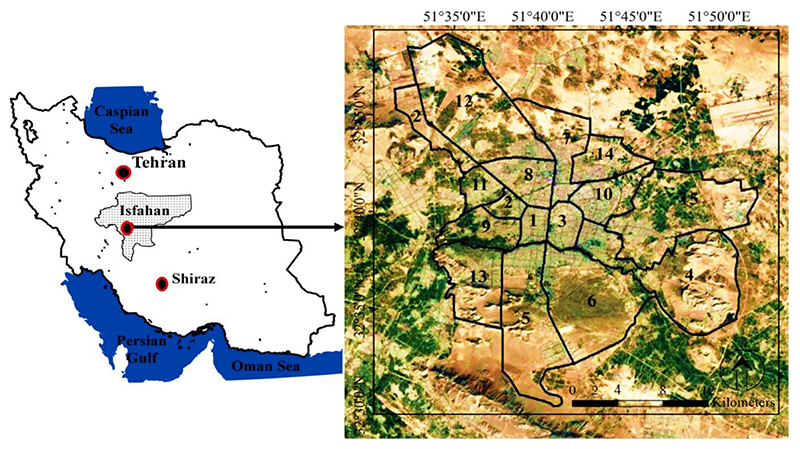
Location of Isfahan metropolis (study area) in Iran and illustration of its 15 management zones.

The study area of Isfahan (between 32°30’00”N to 32°50’00”N and 51°32’30”E to 51°52’00”E) (Figure 1), with a population of 1,961,260 (in 2016) and area of 551 km^2^, is the third largest metropolis of Iran. Isfahan is one of the most important historical, cultural, political, and religious centers of Iran. This city has always been a destination for tourism due to its ancient attractions and architecture. Isfahan has 15 urban-management regions, the locations of which are shown in Figure 1. The study area has a hot and arid climate, with hot summers and cold winters (maximum temperature: 40.6 °C, minimum temperature: –10.6 °C and average annual temperature: 16.7 °C) [48,49]. The annual precipitation is low (average 116.9 mm) and rainfall occurs mostly in wintertime. The Zayandeh-Rood river passes through the middle of the city and the river shores are covered by green space, but for the last several years the river flow has been cut off in summertime. Furthermore, a large number of immigrants migrated from rural areas to Isfahan and it led to the replacement of natural lands with urban landscapes. The green space per capita in Isfahan is 26 m^2^, but the green space in all areas of the city is not evenly distributed and is densely colonized along the Zayandeh-Rood river and the city center [48].

### 2.2 UHI Monitoring

#### 2.2.1 Satellite Images and Pre-Possessing

Images from the Landsat-8 Collection 1 Level-1 were collected from the Earth Explorer website (https://earthexplorer.usgs.gov/). This satellite has two sensors: the operational land imager (OLI) and the thermal infrared sensor (TIRS) with nine spectral bands (spatial resolution of 30 m) and two thermal bands (spatial resolution of 100 m; band 10: 10.60 μm to 11.19 μm, and band 11: 11.50 μm to 12.51 μm) [27]. A total of nine Landsat images taken during summers from 2016 to 2018 were downloaded, which covered Isfahan metropolis in the path and row of 164 and 37, respectively. For each summer, three Landsat images were collected from June, July, and August. For all studied images, the geometric correction was made by in-field control points. Pre-processing corrections, i.e., convert raw digital numbers (DN) to at-satellite brightness temperature for thermal bands and convert raw DN to reflectance with apparent reflectance correction model for multispectral bands, were also applied for all studied images. Then, the image subset tool was used to clip the study boundary from the Landsat image frame. After these pre-processing steps, the images of Isfahan metropolis were prepared for the data processing and subsequent LST analysis. In addition, Isfahan regional maps and Isfahan street maps were used.

#### 2.2.2 Image Processing

**Figure 2:**
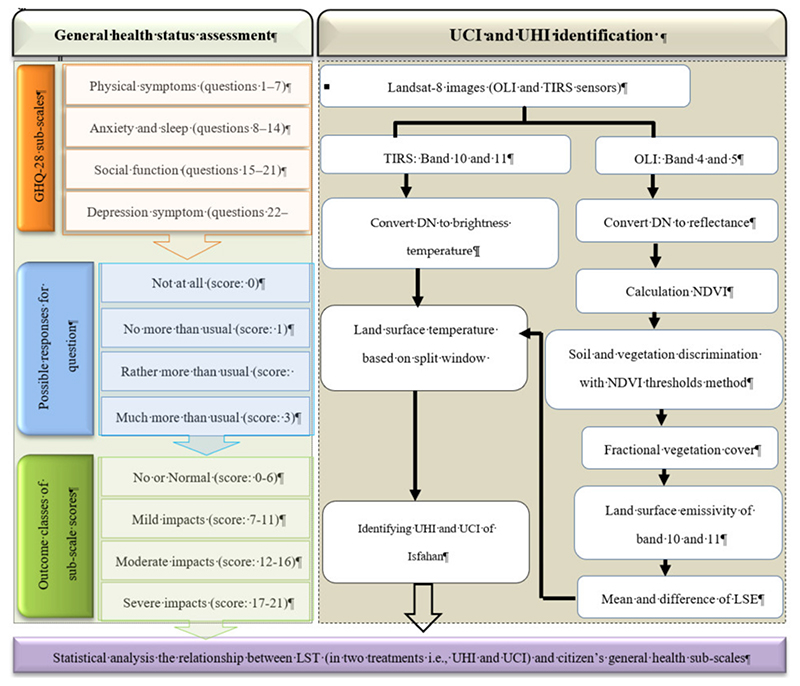
The main flowchart of the study (general health questionnaire-28 (GHQ-28) methodology and retrieving land surface temperature (LST)).

In order to detect the UHI in Isfahan, it was necessary to map the LST of the metropolis. Various approaches have attempted to establish methods for retrieving the LST from optical remote sensing data. The TM and ETM^+^ sensors of Landsat are equipped with only one thermal band, while the TIRS sensor provides two thermal bands. Therefore Landsat-8 is more suited for the split window algorithm (SWA) [27,50,51]. Figure 2 shows the flowchart of the study and overview for performing the SWA. We performed SWA in seven main steps [51,52]: (i) estimation of the top-of-atmospheric (TOA) spectral radiance, (ii) estimation of the brightness temperature (BT), (iii) estimation of the normalized difference vegetation index (NDVI), (iv) estimation of the fractional vegetation cover (FVC), (v) combination of LSE of band 10 and 11, (vi) estimation of land surface temperature (LST), and (vii) classifying hot and cold islands.

As the absolute temperature was not of importance for this study, we instead derived the relative temperature of pixels. Thus, we were only looking for the relative temperature in the studied areas in order to enable identification of the heat and cold islands. In cases where the measurement of the absolute LST is considered, however, the use of Stray Light Correction algorithm for band 11 of Landsat is recommended to increase the accuracy of results [53,54].

#### 2.2.3 Split Window Algorithm

The SWA was applied in order to detect the LST. This algorithm uses BT of two TIRS bands (band 10 and 11 of Landsat imagery), mean, and difference in land surface emissivity (LSE) for estimating the LST of an area. The basic formula for applying this algorithm to Landsat images and measuring LST is as follows [52]:

(1) $$\begin{array}{c} \text{LST}=\text{BTB}10+\text{C}1(\text{BTB}10-\text{BTB}11)+\text{C}2(\text{BTB}10-\text{BTB}11)2+\text{C}0+\\ (\text{C}3+\text{C}4\times \text{W})(1-\text{mLSE})+(\text{C}5+\text{C}6\times \text{W})\times \text{ΔLSE} \end{array}$$

where, BTB_10_ and BTB_10_ are brightness temperature of band 10 and 11, respectively. C_0_ to C_6_ are SWA constant coefficients (Table 1), *m*LSE and ΔLSE are mean and difference of LSE, respectively. W is atmospheric water vapor content (0.05 ≤water vapor content ≤ 0.21 g/cm^2^, calculated based on meteorological data from Isfahan metropolitan area at the time of the satellites overpass).

BT is the microwave radiation radiance traveling upward from the top of Earth’s atmosphere. The transformation process for converting thermal DN values of band 10 and 11 to BT was applied. The TOA spectral radiance is needed for finding BT of an area. BT for both the TIRs bands was calculated as follows:

(2) $$BT=\frac{K_{2}}{Ln_{\left((\frac{K_{2}}{L\lambda }+1\right)}}$$

where, K_1_ and K_2_ are thermal conversion values of band 10 and 11 (Table 1). Lλ is TOA spectral radiance and calculated by Equation (3):

(3) $$L\lambda =R_{M}\times DN_{band\;10/11}+R_{A}$$

where, *R_M_* and *R_A_* are band specific multiplicative rescaling factor and band specific additive rescaling factor, respectively (Table 1). DN is raw digital number of band 10 and 11.

Before obtaining the LST, we first calculated the LSE for the study area in each of the Landsat images. Accordingly, the mean (*m*LSE) and difference (ΔLSE) of LSE band 10 and 11 was calculated. LSE was estimated using NDVI threshold method as follow:

(4) $$LSE=\varepsilon_{soil\;}\times (1-FVC)+\varepsilon_{vegatation\;}\times FVC$$

where, *ε_soil_* and *ε_vegetation_* are emissivity values of soil and vegetation in the corresponding bands, respectively (Table 1). FVC is fractional vegetation cover, which was calculated based on the following equation:

(5) $$FVC=\frac{NDVI-NDVI_{soil\;}}{NDVI_{vegetation\;}-NDVI_{soil\;}}$$

where, *NDVI_soil_* and *NDVI_vegetation_* are reclassified NDVI for soil and vegetation, respectively. NDVI calculated from the red and near-infrared reflectance of Landsat OLI bands [55]. The NDVI values are ranged between −1 and +1, when we divided the NDVI images as −1 ≤ *NDVI_soil_* < 0.2 and 0.2 ≤ *NDVI_vegetation_* ≤ 1 [50]; soil and vegetation were well separated. Finally, the LST maps (in K°) of all the Landsat images of the Isfahan metropolis were generated based of the SWA. It should be noted that all the above steps were done in the TerrSet software package version 18 (formerly IDRISI).

#### 2.2.4 Classification of Heat and Cold Islands

After calculating the temperature, each pixel was assigned to one of the classes according to Table 2. The table was used to identify UHIs and UCIs based on mean standard deviation method. In this regard, the LST classified the map into six thermal levels from the lowest (i.e., cold island) to highest (i.e., heat island) LST. Afterwards, two Boolean maps were prepared from hot (UHI) and cold (UCI) pixels, and four points randomly selected from each of them by X-tool extension in ArcGIS version 9.3 software. Subsequently, these points were used to identify the nearest health centers and questionnaires were completed at the respective health centers. Since the management zones are the determining units in the planning and management of the Isfahan metropolis, the variation of LST at the zone level was also analyzed. This was done with the zone statistics tool in ArcGIS. It should be noted that the Isfahan metropolis has 15 administrative-management zones, the distribution of which was shown in Figure 1 (www.isfahan.ir).

### 2.3 Health Assessment of the Study Subjects

After identifying the hot and cold islands and ranking Isfahan’s management zones in terms of LST, four healthcare centers located in UCIs and four centers located in UHIs were selected. Then, at each healthcare center 100 people were randomly selected to complete the GHQ-28 in August 2019. In Isfahan, there are healthcare centers that provide various services, e.g., vaccination, mother and child-care, annual censuses, family planning services, occupational medicine and occupational health services, and environmental health services. Each of these centers cover a population of about 5000 and most people go to them for healthcare monitoring (http://dh.zaums.ac.ir/21258.page). Healthcare centers record all medical and personal information of covered population in the form of lists. We used these lists to randomly select participants. Therefore, GHQ-28 assessed the health status of 400 subjects in the UHIs and 400 subjects in the UCIs.

It is worth noting that here the health status of the subjects affected by UHIs and UCIs was for the first time assessed using the GHQ. The GHQ is one of the most commonly used tools for assessing physical and mental health worldwide and has multiple, self-administered constructs. The questionnaire was developed by Goldberg [40]. This questionnaire is one of the most well-known screening tools for mental disorders as well [57]. Several methods are possible to score the GHQ-28. The questionnaire was scored by the Likert method [58,59]. The GHQ-28 has four sub-scales, each of which has seven questions. The sub-scales of this questionnaire are: physical (somatic) symptoms (questions 1–7); anxiety and sleep (questions 8–14); social function (questions 15–21), and depression symptoms (questions 22–28) [40,41]. Each question is accompanied by four possible responses, which can be scored from 0 to 3, i.e., *Not at all* (score: 0), *No more than usual* (score: 1), *Rather more than usual* (score: 2), *and Much more than usual* (score: 3). Therefore, the total score of each questionnaire can be between 0 and 84 (28 × 3 = 84). In this method, the total scores are given in four outcome classes, i.e., (1) No or Normal (total score: 0–22), (2) Mild impacts (total score: 23–40), (3) Moderate impacts (total score: 41–60), and Severe impacts (total score: 61–84). It should be noted that a total score more than 22 is considered as the threshold for the presence of distress [41]. This classification was also defined for sub-scale scores. The total score of each sub-scale can be allocated in four outcome classes, i.e., (1) No or Normal (score: 0–6), (2) Mild impacts (score: 7–11), (3) Moderate impacts (score: 12–16) and Severe impacts (score: 17–21).

To evaluate the validity and reliability of this questionnaire, multiple studies have been conducted abroad and internally [58–61]. It is also worth mentioning that the quality of this questionnaire has been proven in previous studies in Iran [59,62,63]. Test-retest reliability has been reported to be high (0.78 to 0.90) [42] and interrater and intrarater reliability have both been shown to be excellent (Cronbach’s α 0.90–0.95) [44]. In a study conducted by Askary-Ashtiani et al. [64] on the 28-question form of this questionnaire, sensitivity was 80%, specificity 99%, criterion validity coefficient 0.78 and Cronbach’s alpha was 0.97. A high internal consistency has also been reported [44]. The GHQ-28 correlates well with the Hospital Depression and Anxiety Scale (HADS) [43] and other measures of depression [42].

### 2.4 Statistical Analysis

One-way ANOVA test was used to compare the average LST of the studied years and detect any significant difference with a 95% confidence level (**P** = 0.05). It should be noted that we had nine images (three images per year) and that LST was estimated for each pixel of them. For each pixel, the annual mean of the studied years (2016, 2017, and 2018) was calculated separately and three corresponding data sets were obtained at pixel level. Then, one-way ANOVA test was applied to compare the mean between these three groups. This test also was used for comparing the average temperature between Isfahan management zones. We applied Mann–Whitney test (at 95% confidence level) to compare general health sub-scales (i.e., physical health, anxiety and sleep, social function, and depression) between citizens in UHI and UCI and determine the significant differences between the treatments. Statistical analysis and graph drawing were done with Microsoft Excel 2010 and the SPSS version 16.

## 3 Results and Discussion

### 3.1 Temporal Variation of LST

**Figure 3:**
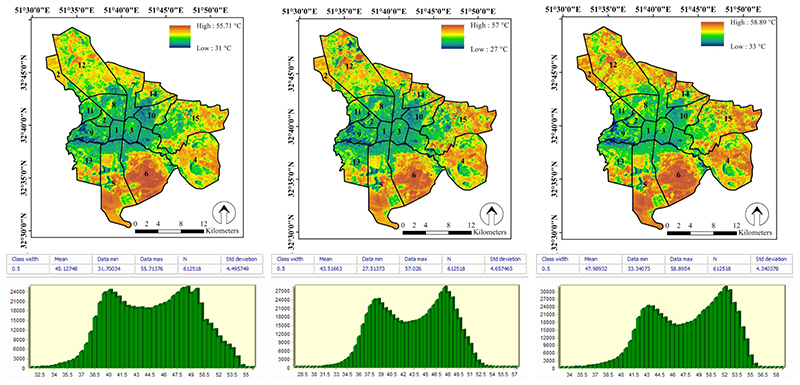
Mean LST maps of 2016, 2017, and 2018 (n = 3) and summary statistics of them (Vertical axis: No. of pixel, Horizontal axis: temperature in °C).

LST monitoring was carried out on the summer of 2016, 2017, and 2018 with nine images of the Landsat satellite (three images for each year) in Isfahan metropolis based on the SWA. Figure 3 shows the mean LST maps of 2016, 2017 and 2018, and summary statistics of LST. Accordingly, the minimum and maximum LST for the years 2016, 2017, and 2018 were 31–55.7 °C, 27–57 °C and 33–58.9 °C, respectively The mean LST during these years was 45.1, 43.5, and 48 °C, respectively. Although along the studied years the LST was varied (2018 and 2017 had the highest and lowest LST, respectively), the mean comparison test showed that there was no significant difference (*P* > 0.05) between the LST in the studied years. The lack of significant difference between the mean LST in the studied years could be due to the short duration of the study period (three years) and the lack of changes in the factors affecting the ground surface temperature in Isfahan metropolis. One of the most important factors affecting urban LST is land use composition, e.g., green spaces and man-made surfaces [5,14,65,66]. In this respect in Shirani-bidabadi et al. [56], where the Isfahan green spaces and LST interactions over a 17-year period (1999-2016) were investigated, significant differences were observed between the studied years in terms of LST. Madanian et al. [49] also examined the effect of land use characteristics on LST in Isfahan metropolis over a 30-year period (1985-2015). Their results showed that land use change had a significant effect on Isfahan land surface temperature during this period. Thus, in general, it can be assumed that the surface temperature during the studied three-year period kept approximately the same pattern. We chose this period to select cold and heat islands for distribution of health questionnaires in the year 2019.

### 3.2 Spatial Variation of LST

**Figure 4:**
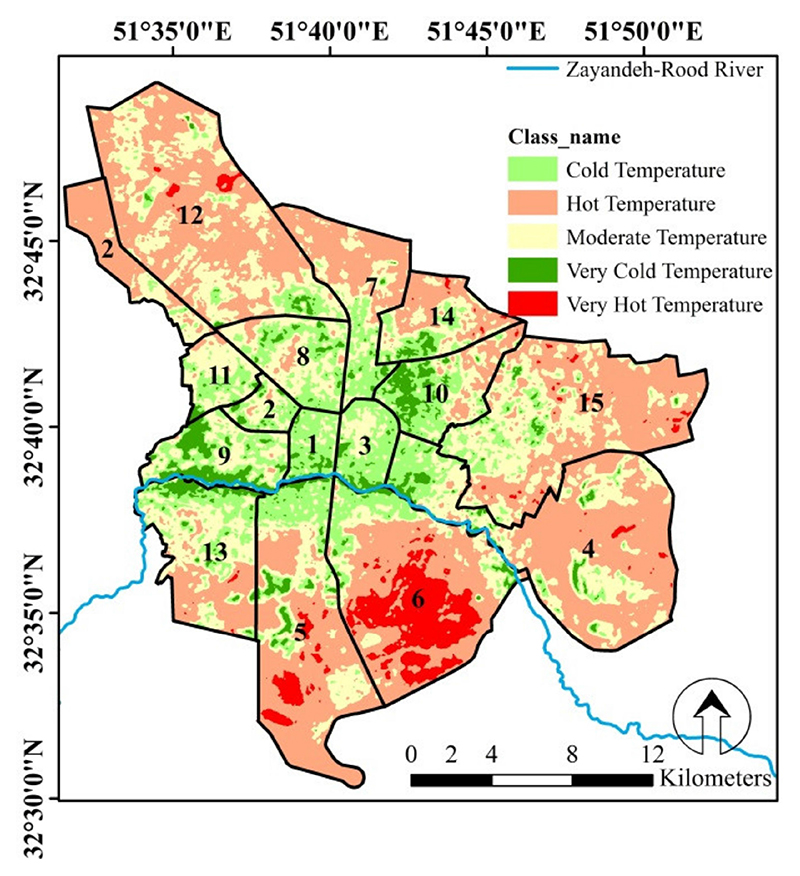
LST classified map of Isfahan based on mean-standard deviation method (n = 9) in the study period.

Figure 4 shows the LST map of the Isfahan metropolis in five classes: (1) very cold, (2) cold, (3) moderate, (4) hot, and (5) very hot temperature. These classes covered 4.9%, 16.5%, 30.1%, 43.8% and 4.7% of the Isfahan metropolis area, respectively. We considered the extremes, “very cold temperature class” and “very hot temperature class”, as UCI and UHI, respectively. This map was produced from the mean of LST maps in the years 2016, 2017, and 2018. Accordingly, the central parts of the Isfahan metropolis exhibited the highest portion of cold and very cold classes. Whereas the northern, eastern, and southern parts of Isfahan exhibited higher LST and were classified as hot and very hot. While the highest population density and the highest traffic volume are focused in the central parts of the city, there are some contributing factors that can maintain temperature balance in the city center, e.g., the flow of Zayandeh-Rood river and the greenery congestion around the river [56]. In support of this finding, Madanian et al. [49] also acknowledged that the Zayandeh-Rood river and its surrounding greenery play a vital role in controlling Isfahan’s LST. The Zayandeh-Rood river flows through the center of Isfahan and is surrounded by dense parks and greenery, in particular trees prevent the increasing earth temperature through shading. Peng et al. [29] suggested that green spaces play a significant role in mitigating the LST in the Beijing metropolitan area, especially if the green space patches are integrated and wide, their cooling effect will increase. Park et al. [66] investigated the influence of green spaces on UHIs in Seoul, South Korea. They found that green space patches with an area over 300 m^2^ and 650 m^2^ can lower the LST by 1 and 2 °C, respectively. It is worth noting that despite the importance of green space in regulating the earth’s temperature, the results of Shirani-bidabadi et al. [56] showed that the green space of the Isfahan metropolis had decreased in 2016 as compared to in 1999, and this is a cause for concern.

**Figure 5:**
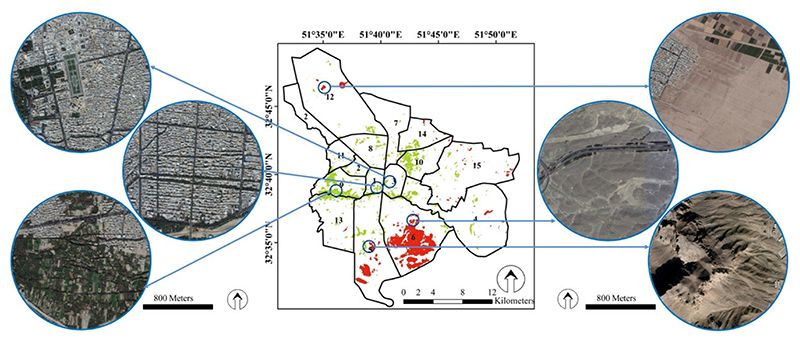
Map of heat and cold islands in Isfahan metropolis and zoomed examples by Google Earth images.

In order to gain a better understanding in the location of the heat and cold islands in Isfahan, more details of the city land covers are shown in Figure 5. This figure confirms that the greenery around the river or on the outskirts of the streets led to the creation of cold islands.

Isfahan is located in a hot and arid region and its suburban area is covered by bare and rocky lands [49]. This land cover absorbs and reflects more sunlight and leads to a higher surface temperature in suburban areas [67]. In confirmation of this, Lazzarini et al. [68] stated that cities located in hot and arid regions exposed higher surface temperatures in their suburban as opposed to central parts.

Most of the heat islands are located in the northern, eastern, and southern parts of Isfahan. Figure 5 also confirms that these parts have a lack of, or poor, vegetation cover and are often covered by barren rocky terrain as well as man-made surfaces. It is worth mentioning that some human activities led to an increase in LST, e.g., a military-based area (in the south east), a passenger transport terminal (in the south west), and an increase in the activities of thermal power plants and petrochemical complexes and an oil refinery in the western part of Isfahan [56].

**Figure 6:**
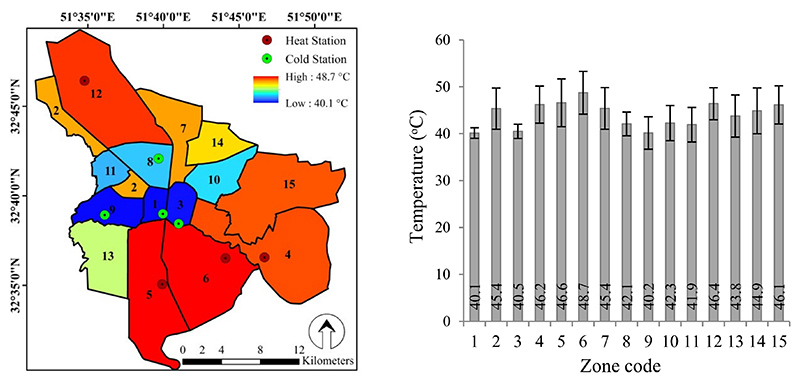
Map of the average LST in the management zones and location of selected heat and cold stations (left), and statistical summary chart (average and standard deviation: n = 9) of LST in the management zones (right).

This study investigated Isfahan LST and the determination of UHI and UCI in the years 2016, 2017, and 2018 to identify suitable healthcare centers for completing GHQ-28 in 2019. Isfahan city management is based on its 15 zones. Therefore, the environmental conditions, services, and available facilities in each zone have a direct impact on the health of its residents. In Figure 6, Isfahan management zones were ranked based on the mean LST over the three-year period. Accordingly, zones 1 and 6 exhibited the coldest (40.1 °C) and hottest (48.7 °C) LSTs, with a temperature difference of 8.6 °C. A significant difference (*P* < 0.05) was also observed between the surface temperatures of these zones. We identified four of the hottest zones (UHI) and four of the coldest zones (UCI) in Isfahan based on the results shown in Figure 6 and selected a healthcare center in each zone to complete the GHQ. Therefore zones 1, 3, 8, and 9 were assigned to the UCI treatment, and zones 4, 5, 6, and 12 were considered as UHI treatment.

### 3.3 Relation Between UHI and General Health Sub-Scales

UHIs are one of the most important factors affecting the health and quality of citizen’s life [32,35,36]. But it is necessary to prove this hypothesis accurate with statistical analysis. Therefore, the usage of a questionnaire can be a rapid, easy, and low-cost way to test this hypothesis. We used the GHQ-28 to analyze the effects of UHIs/UCIs on citizens’ health status. In this respect, a fundamental difference between this and previous related studies [30,31,69,70] is that we assessed the general health status of citizens based on a questionnaire (GHQ-28), while in the earlier studies the health data were obtained through hospitalization statistics, mortality rate, etc.

To sum up, we monitored UHIs and UCIs over a three-year period (2016–2018) in the Isfahan metropolitan area and the relationship between citizens’ general health status and the urban surface temperature was determined using a GHQ-28 based on statistical analysis. Scoring was done based on GHQ-28 and the results were compared in two groups, i.e., citizens in UHIs and in UCIs. Considering the statistical analysis of the GHQ-28 in the four sub-scales, i.e., (1) physical health, (2) anxiety and sleep, (3) social functioning and (4) depression, the results are presented in Table 3.

The results of statistical analysis (Mann–Whitney test) showed that the physical health of citizens in both UHIs and UCIs was not significantly different (*P* > 0.05), but there was significant difference between the two groups of citizen in terms of: (1) social function, (2) depression, and (3) anxiety and sleep (*P* < 0.05). In other words, according to the results presented in the table, it can be stated that the citizens in UHIs have shown more severe responses in terms of social function, depression, and anxiety and sleep. These are important health indicators and underline the importance of paying attention to UHIs and the role of city planning.

Consistent with our findings, the studies of Huynen et al. [31] and Gosling et al. [30] found that UHIs strongly affect citizens’ health indices, even leading to higher mortality rates in these areas. Similarly, Jagai et al. [69] studied heat stress diseases in urban and surrounding areas. Their results showed that high temperature had a significant effect on the hospitalization rate. Psychological and social health issues associated to UHIs, such as depression and the restriction of social activities, were also reported by Wong et al. [36]. In another study, Chan et al. [70] reported a positive relationship between ambient temperatures and mental disorder hospitalizations in the Hong Kong special administrative region. Wong et al. [71] also examined the effects of UHI on social, physical, and mental health indices of citizen in Greater Kuala Lumpur, Malaysia. Their results stressed the importance of the health effects of UHIs. They found that the urban temperature can alter the rate of psychological distresses, e.g., anxiety, depression, and aggressive behaviors. They also reported that the levels of socialization were reduced and prominent physical health impacts such as heat exhaustion, respiratory problems, and heat stroke.

When bringing all these studies together with ours, we can conclude that the role of the UHIs in creating a health risk to citizens is substantial. Moreover, the important point is that the present results show that the non-physical sub-scale of the citizens’ health (i.e., anxiety and sleep, social functioning, and depression) were affected by UHIs. Conversely, the physical health index of the citizens in the UHI and UCI treatments had a similar pattern. Overall, 92.7 % and 91.2 % of citizens showed Moderate and severe responses to physical health in UHI and UCI treatments, respectively (Table 3), and this shows that the heat has generally disrupted the physical health of citizens. The lack of a significant difference in physical health between citizens in the two treatments could be due to the fact that it is easier to cope with and modify the effects of heat. For example, wearing cooler clothing, using cooling equipment, and going into the shade can reduce the physical effects of heat. However, non-physical dimensions of heat increase the anxiety, concern, and social functioning of citizens and make it more difficult to cope with and is a concern.

As a final remark, although numerous studies analyzed urban heat islands and their effects on human general health status, so far, no study has been conducted using GHQ-28 and this study is innovative in this respect. However, the efficacy and utility of using GHQ approaches to assess and quantify the health status of communities is well known. For example, Dzhambov et al. [72] investigated the relationships between noise and air pollution in the mental illnesses of 720 students (18–35 years) in Bulgaria. In their study, general health status was assessed by GHQ, and they cited the GHQ method as a rapid, economical, and practical approach for assessing general health status. Other studies also used the GHQ approach to analyze the relationship between citizens’ health and environmental pollution, e.g., road traffic noise and persistent organic pollutants [73,74]. Accordingly, combining GHQ approaches together with remote sensing analysis, as demonstrated here, proved to be an effective and cheap method to monitor the life quality of citizens.

## 4 Conclusions

Due to the adverse effects of urban heat islands (UHIs) on human health, energy consumption, and environment components, evaluating the distribution patterns of this phenomenon can play a significant role in mitigating UHI effects, especially in metropolises. The aim of this study was to investigate the effect of UHIs on community general health status using a remote sensing and field survey approach in the Isfahan metropolis, Iran. UHI monitoring of the Isfahan metropolis was conducted by Landsat-8 images and split window algorithm for three years (2016, 2017, and 2018), and the health status of citizens in two treatments (UHI and UCI) evaluated by GHQ-28. Our most important findings are listed as follows:

i) The average LST during the study period was 45.5 ± 2.3 °C, and no significant difference (*P* > 0.05) was found between these years. This suggests that the LST pattern was almost identical during the study period and that the citizens were exposed to UHI and UCI during the period.
ii) UCI and UHI extremes covered 4.9 % and 4.7 % of the Isfahan metropolitan area, respectively, and the Zayandeh-Rood river and the surrounding greenery played an important and vital role in moderating the surface temperature in the densest and most trafficked part of the city.
iii) A total of 800 citizens completed the GHQ-28 in both UCI and UHI treatments. The results showed that non-physical sub-scales of citizens’ health (i.e., anxiety and sleep, social functioning, and depression) in UHIs were more severely affected.

Our results demonstrated that suburban areas of the Isfahan metropolis, rather than the central parts, are more influenced by UHI effects. The suburbs of Isfahan are characterized by poor vegetation cover and the barren rocky terrain and man-made surfaces. Therefore, it could be a suggestion for city administrators to consider greener space and health facilities for citizens in hot management zones. More interventions should be developed to impart knowledge and encourage the citizens to engage in measures to counter the effects of UHIs and maintain optimal well-being. The factors influencing health impacts found in this study can and should inform policy and public health responses to mitigate UHI effects.

This study exemplified that the combination of optical remote sensing techniques and ground-based survey can play an important role in monitoring citizens’ health and optimizing urban management, providing valuable information at a low cost and in a short time. The survey GHQ-28 is easy, rapid, and low-cost, and is capable of assessing the general health status of citizens’ and providing useful information. It is suggested to conduct similar studies on other metropolises and evaluate the role of UHIs in increasing environmental risk to citizens’ health.

## Figures and Tables

**Table 1:** Details of the data used to run the SWA on Landsat-8 images [51,52].

Input Name	Band 10	Band 11	Values
	**Emissivity**		
^ε^soil	0.971	0.977	
^ε^vegetation	0.987	0.989	
	**Thermal constant value**		
**K_1_**	774.8853	480.8883	
**K_2_**	1321.0789	1201.1442	
	**Rescaling Factor**		
**R_M_**	0.0003342	0.0003342	
**R_A_**	0.1000000	0.1000000	
	**SWA constant coefficients**		
**C_0_**	-	-	–0.268
**C_1_**	-	-	1.378
**C_2_**	-	-	0.183
**C_3_**	-	-	54.300
**C_4_**		-	–2.238
**C_5_**	-	-	–129.200
**C_6_**	-	-	16.400

**Table 2:** LST classification of the studied images [56].

Class Name	Class Range
Very cold temperature	*T* ≤ Tmean – 1.5std
Cold temperature	Tmean – 1.5std < *T* ≤ *Tmean* + *std*
Moderate temperature	Tmean – std < *T* ≤ *Tmean* + *std*
Hot temperature	Tmean + std < *T* ≤ *Tmean* + 1.5std
Very hot temperature	T > *Tmean* + 1.5std

**Table 3:** Summary of GHQ-28 results and statistical comparing of GHQ-28 sub-scales (i.e., physical health, social function, depression, and anxiety and sleep) in two groups of citizen (urban heat island (UHI) and urban cold island (UCI)) based on Mann–Whitney test (at 95% confidence level).

Responses	Citizens in UHI		Citizens in UCI		*p* Value
	Number	Percentage Number	Percentage		
	physical health				
Mild	29	7.3	32	8.1	
Moderate	318	80.7	320	80.8	0.102
Severe	47	12.0	44	11.1	
	**Social Function**				
Mild	18	4.6	20	5.1	
Moderate	332	84.3	364	91.9	0.007
Severe	44	11.2	12	3	
	**Depression**				
Mild	324	81.4	366	92.4	
Moderate	60	15.1	18	4.5	0.002
Severe	14	3.5	12	3	
	**Anxiety and Sleep**				
Mild	216	54.3	230	58.4	
Moderate	124	32.2	142	36	0.012
Severe	58	14.6	22	5.6	
